# Pre-clinical characterization of PKC412, a multi-kinase inhibitor, against colorectal cancer cells

**DOI:** 10.18632/oncotarget.12802

**Published:** 2016-10-21

**Authors:** Jian-Ping Li, Zhi-Jun Huang, Xing-Sheng Lu, Yi-Chan Zhou, Yun Shao, Xiao-Pu He, Su-Rong Chen, Dong-Dong Wang, Li-Sen Qin, Wei-Hao Sun

**Affiliations:** ^1^ Department of Geriatric Gastroenterology, The First Affiliated Hospital of Nanjing Medical University, Nanjing, China; ^2^ Department of Oncology, Yancheng Fist People's Hospital, Yancheng, China; ^3^ Department of Surgery, The First Affiliated Hospital of Soochow University, Suzhou, China; ^4^ Department of Surgery, Yancheng Fist People's Hospital, Yancheng, China; ^5^ Department of Hepatobiliary Surgery, Suzhou Municipal Hospital, Suzhou, China; ^6^ Department of Neurosurgery, Yancheng Pavilion Lake District People's Hospital, Yancheng, China

**Keywords:** colorectal cancer (CRC), PKC412, AKT, Bcl-2, receptor tyrosine kinases

## Abstract

The potential effect of PKC412, a small molecular multi-kinase inhibitor, in colorectal cancer (CRC) cells was evaluated here. We showed that PKC412 was cytotoxic and anti-proliferative against CRC cell lines (HT-29, HCT-116, HT-15 and DLD-1) and primary CRC cells. PKC412 provoked caspase-dependent apoptotic death, and induced G2-M arrest in the CRC cells. AKT activation was inhibited by PKC412 in CRC cells. Reversely, expression of constitutively-active AKT1 (CA-AKT1) decreased the PKC412's cytotoxicity against HT-29 cells. We propose that Bcl-2 could be a primary resistance factor of PKC412. ABT-737, a Bcl-2 inhibitor, or Bcl-2 siRNA knockdown, dramatically potentiated PKC412's lethality against CRC cells. Forced Bcl-2 over-expression, on the other hand, attenuated PKC412's cytotoxicity. Significantly, PKC412 oral administration suppressed AKT activation and inhibited HT-29 tumor growth in nude mice. Mice survival was also improved with PKC412 administration. These results indicate that PKC412 may have potential value for CRC treatment.

## INTRODUCTION

Surgical resection of early-defined tumors and chemotherapies are applied for colorectal cancer (CRC) treatment, yet they have failed to significantly improve patients' prognosis [1-3]. The five-year overall survival for the advanced and/or metastatic CRC patients is not satisfactory [1-3]. Considerable efforts have been spent to explore novel chemo-preventive agents against CRC [4, 5]. It has also been our research focus in recent years [6].

PKC412 (*N*-benzoylstaurosporine) was originally developed as a small-molecular inhibitor of protein kinase C (PKC) [7]. Later on, it was demonstrated that this compound could also inhibit multiple class III tyrosine kinases, including vascular endothelial growth factor receptor 2 (VEGFR-2), c-kit, platelet-derived growth factor receptor α (PDGFR-α), and PDGFR-β [8]. Considering that most of these kinases were dysregulated and/or over-activated in CRC [5, 9], we here tested the anti-cancer activity of this multi-kinase inhibitor in pre-clinical CRC models [6].

## RESULTS

### PKC412 inhibits CRC cell survival and proliferation

**Figure 1:**
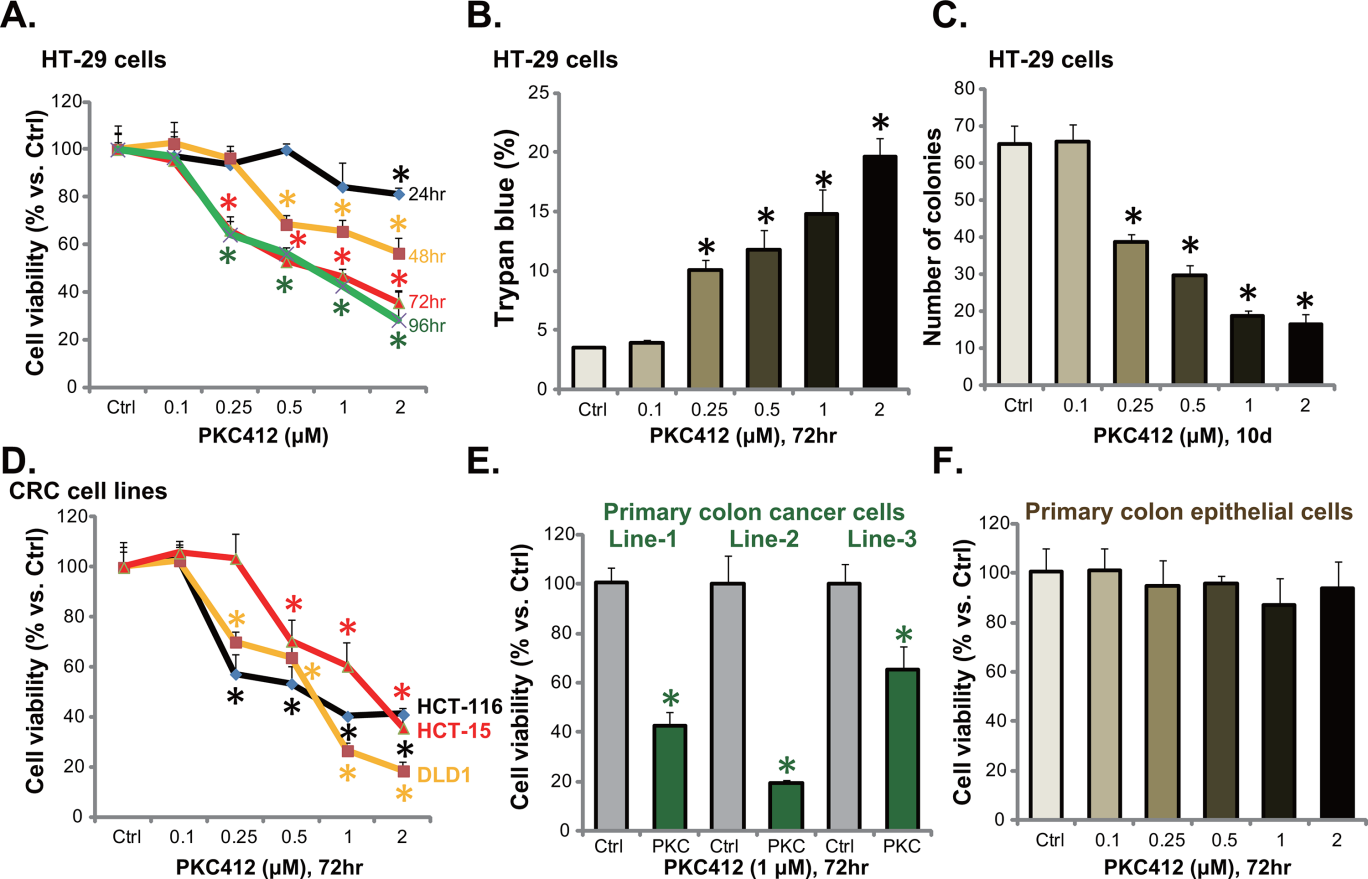
PKC412 inhibits CRC cell survival and proliferation CRC cell lines (HT-29, HCT-116, HCT-15 and DLD1), the primary human colon cancer cells (thee lines, Line-1/-2/-3) or primary human colon epithelial cells were treated with applied concentrations of PKC412 (0.25-2 μM), cells were further cultured for indicated time, and cell survival was tested by MTT assay (**A**, **D**, **E** and **F**); Cell death was tested by the trypan blue staining assay (**B**, for HT-29 cells); Cell proliferation was examined by the clonogenicity assay (**C**, for HT-29 cells). Experiments in this figure were repeated three times, and similar results were obtained. Error bars indicate standard deviation (SD). *p<0.05 vs. untreated control (“Ctrl”) group.

First, HT-29 CRC cells were cultured and treated with different concentrations of PKC412. MTT assay was applied to test cell viability, and results in Figure 1A demonstrated that PKC412 inhibited HT-29 cell survival in time- and dose-dependent manners. Consequently, HT-29 cell death, or trypan blue positive staining, was increased with PKC412 (0.25-2 μM) treatment (Figure 1B). Next, clonogenicity assay was applied to test the effect of PKC412 on HT-29 cell proliferation. As demonstrated, 0.25-2 μM of PKC412 dramatically decreased the number of proliferative HT-29 cell colonies (Figure 1C). The potential effect of PKC412 on other CRC cells were also examined. MTT assay results in Figure 1D demonstrated that this multi-kinase inhibitor [8] was also cytotoxic to three other CRC cell lines: HCT-116, HCT-15 and DLD-1. Significantly, when adding to the primary human colon cancer cells, PKC412 also decreased survival of the primary cells (Figure 1E). A total of three primary colon cancer cell lines were established, and PKC412 was cytotoxic to all of them (Figure 1E). Intriguingly, the primary human colon epithelial cells (non-cancer cells) were resistant to the same PKC412 treatment (Figure 1F). We repeated these experiments in two other primary colon epithelial cell lines (derived from other two patients), and similar results were obtained (Data not shown). These results show that PKC412 inhibits CRC cell survival and proliferation.

### PKC412 provokes apoptosis in CRC cells

**Figure 2:**
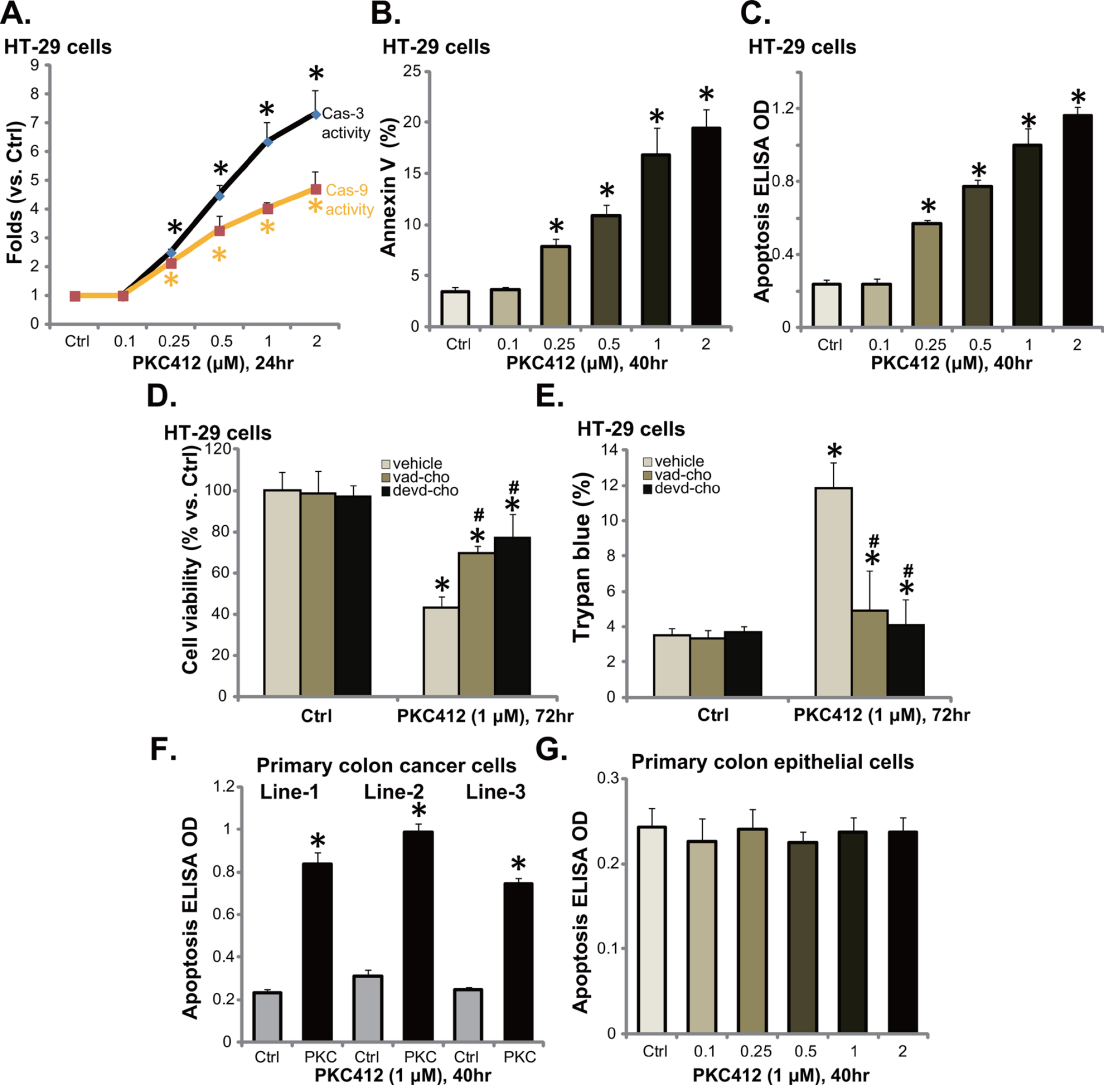
PKC412 provokes apoptosis in CRC cells HT-29 cells, primary human colon cancer cells, or human colon epithelial cells were treated with designated concentrations of PKC412, cells were further cultured, and cell apoptosis was tested by described assays (**A-C**, **F** and **G**). The effect of pan caspase inhibitor z-VAD-CHO (“vad-cho”, 50 μM, 1 hour pre-treatment) or the caspase-3 specific inhibitor z-DEVD-CHO (“devd-cho”, 50 μM, 1 hour pre-treatment) on PKC412 (1 μM)-induced HT-29 cytotoxicity was tested (**D** and **E**). Experiments were repeated three times in this figure, and similar results were obtained. Error bars indicate SD. *p<0.05 vs. untreated control (“Ctrl”) group. ^#^*p*<0.05 vs. PKC412 only group (D and E).

Via applying the methods described in our previous studies [6, 10, 11], we tested apoptosis in PKC412-treatd CRC cells. PKC412, at 0.25-2 μM, dose-dependently increased activity of caspase-3 and caspase-9 in HT-29 cells (Figure 2A). Further, the percentage of Annexin V positive cells was increased following PKC412 treatment (0.25-2 μM) (Figure 2B). Same PKC412 treatment also dose-dependently increased the apoptosis ELISA OD of HT-29 cells (Figure 2C). These results confirmed that PKC412 provoked apoptosis in HT-29 cells. Similar pro-apoptosis results by PKC412 were also seen in other CRC cell lines: HCT-116, HCT-15 and DLD1 (Data not shown).

Next, the pan caspase inhibitor z-VAD-CHO and the caspase-3 specific inhibitor z-DEVD-CHO, were applied to inhibit apoptosis activation in HT-29 cells. MTT assay results in Figure 2D and trypan blue results in Figure 2E demonstrated that pre-treatment with the caspase inhibitors largely attenuated PKC412-induced HT-29 cell viability reduction and cell death. These results suggest that PKC412 induces caspase-dependent apoptosis death of CRC cells. The apoptosis ELISA assay results in Figure 2F showed that PKC412 (1 μM) also induced apoptosis activation in all three lines of primary human colon cancer cells. Yet, no significant apoptosis induction was observed in PKC412 (0.25-2 μM)-treated primary colon epithelial cells (Figure 2G).

### PKC412 disturbs CRC cell cycle progression

**Figure 3:**
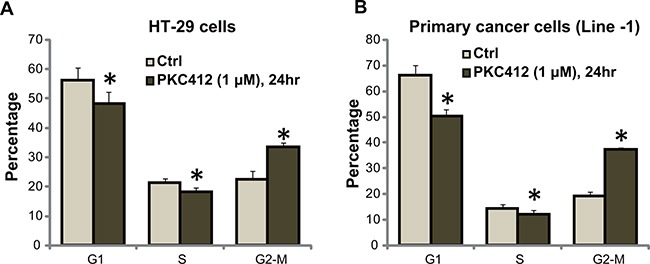
PKC412 disturbs CRC cell cycle progression HT-29 cells **A.** or the primary human colon cancer cells (Line-1) **B.** were treated with or without PKC412 (1 μM) for 24 hours, cell cycle distribution was tested. Experiments in this figure were repeated three times, and similar results were obtained. Error bars indicate SD. *p<0.05 vs. untreated control (“Ctrl”) group.

We also tested the potential activity of PKC412 on cell cycle progression using the described FACS method [6]. As shown in Figure 3A, in HT-29 cells, following treatment of PKC412 (1 μM), the percentages of G1 and S phase cells were both decreased. Correspondingly, the G2-M phase cell percentage was increased (Figure 3A). We performed the same experiments in primary human colon cancer cells, similar G2-M arrest was noticed following PKC412 treatment (Line-1, Figure 3B, and Line-2/-3, data not shown). Therefore, PKC412 induces G2-M arrest in CRC cells [6].

### PKC412 in-activates AKT in CRC cells

**Figure 4:**
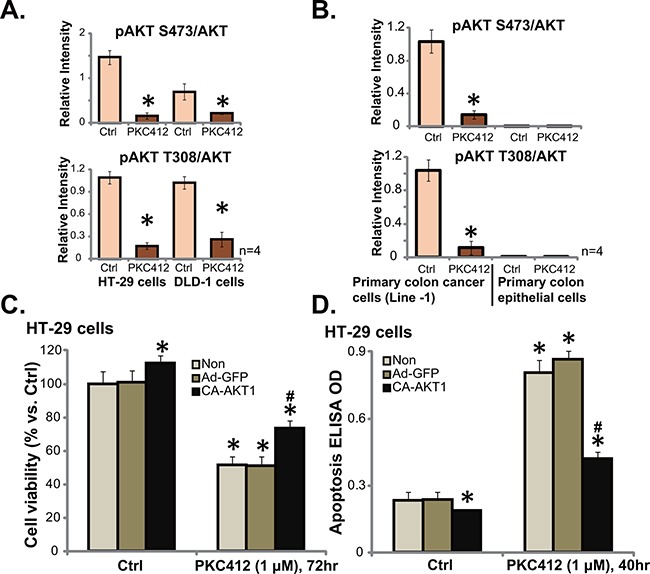
PKC412 in-activates AKT in CRC cells HT-29 cells (**A**, left), DLD-1 cells (A, right), primary human colon cancer cells (Line-1, **B**, left) or colon epithelial cells (**B**, right) were treated with PKC412 (“PKC”, 1 μM) for 6 hours, cells were further cultured, AKT1 (p- and regular) expression was tested by Western blot assay, and their relative intensity (vs. AKT1) was quantified. Stable HT-29 cells, expressing empty vector (“Ad-GFP”), or constitutively active-AKT1 (CA-AKT1), were treated with PKC412 (1 μM) for applied hours, cell survival was tested by MTT assay (**C**), and cell apoptosis was tested by ELISA assay (**D**). “Non” stands for un-transfected parental cells (C and D). Error bars indicate SD. *p<0.05 vs. untreated control (“Ctrl”) group (C and D). ^#^*p*<0.05 vs. “Ad-GFP” group (C and D).

Over-activation of multiple kinases (PDGFR, c-Kit, VEGFR *etc*) in CRC causes AKT dysregulation, which promotes cancer cell progression and apoptosis-resistance [12]. PKC412 is a multiple-kinase inhibitor [8, 13], we therefore analyzed AKT signaling in PKC412-treated cells. Quantified Western blot results in Figure 4A showed that, in both HT-29 cells and DLD-1 cells, treatment with PKC412 (1 μM) resulted in significant AKT inhibition, or p-AKT (at Ser-473 and Thr-308) decrease. Similarly, PKC412 in-activated AKT in primary human colon cancer cells (Line-1, Figure 4B, and Line-2/-3, data not shown). On the other hand, the basal AKT activation (p-AKT Ser-473 and Thr-308) was extremely low in primary colon epithelial cells (Figure 4B), which might explain why PKC412 was not cytotoxic to these non-cancerous cells. To study the link between AKT in-activation and PKC412-induced cytotoxicity, we utilized the constitutively-active AKT1 (CA-AKT1) [6]. As expected, expression of CA-AKT1 in HT-29 cells restored AKT activation in PKC412-treated cells (Data not shown). Remarkably, PKC412's cytotoxicity, tested by viability reduction (Figure 4C) and apoptosis induction (Figure 4D), was significantly attenuated in CA-AKT1-expressing HT-29 cells. Collectively, these results show that PKC412 in-activates AKT in CRC cells, which is responsible for subsequent cell death.

### Bcl-2 is a primary resistance factor of PKC412 in CRC cells

**Figure 5:**
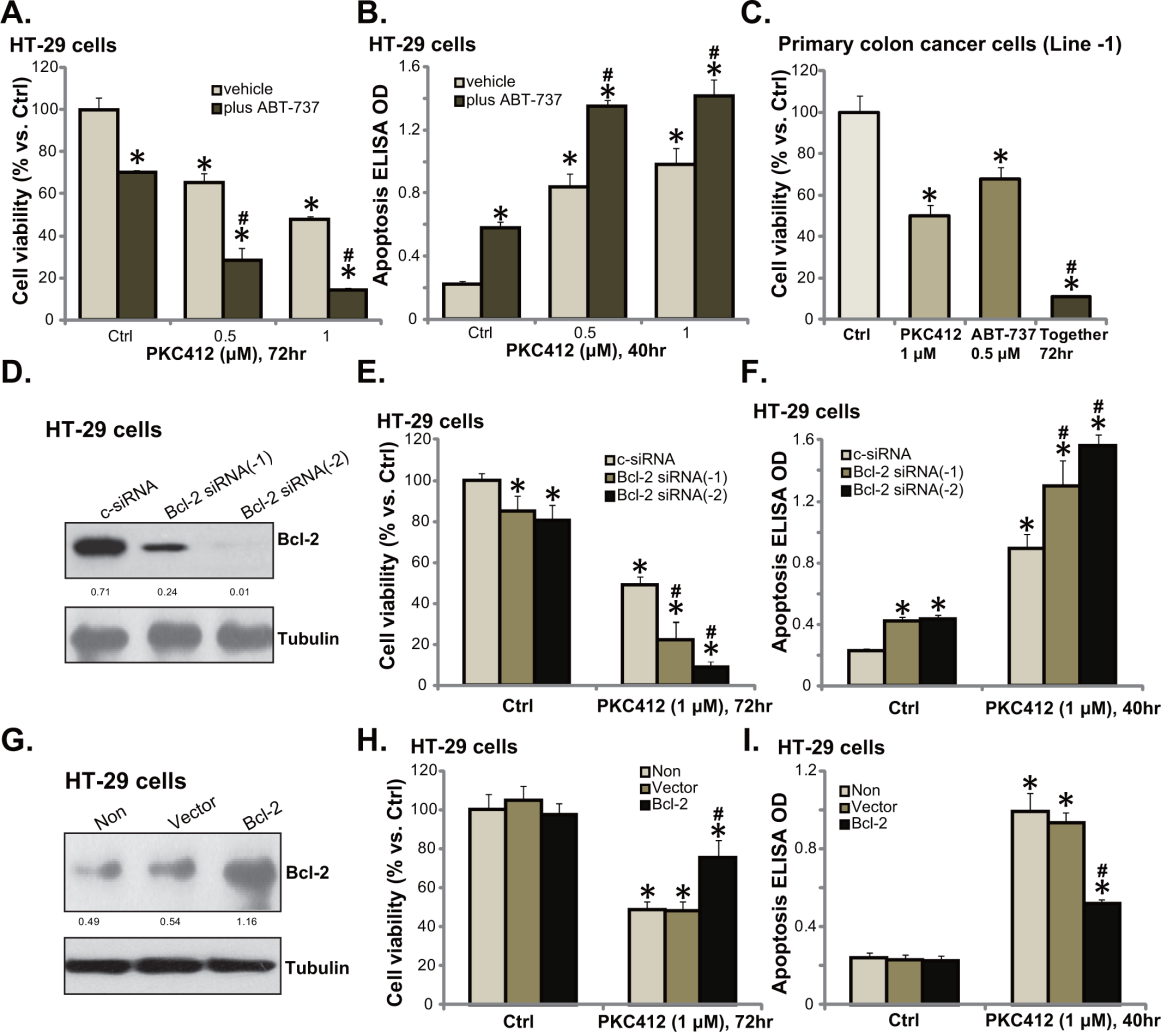
Bcl-2 is a primary resistance factor of PKC412 in CRC cells HT-29 cells (**A** and **B**) or primary human colon cancer cells (Line-1, **C**) were treated with applied concentrations of PKC412 (0.5/1 μM), or plus ABT-737 (0.5 μM), cell survival (MTT assay) and apoptosis (Histone DNA ELISA assay) were tested. HT-29 cells were transfected with control scramble siRNA (“c-siRNA”) or Bcl-2 siRNA (“−1/−2”) for 36 hours, expression of listed proteins was examined by Western blot assay (**D**); These cells were also treated with or without PKC412 (1 μM), cell survival (**E**) and apoptosis (**F**) were tested. Stable HT-29 cells, expressing Ad-GFP (“Vector”) or Bcl-2 plasmid (see Western blot data in **G**), were treated with PKC412 (1 μM) for applied time, cell survival (**H**) and apoptosis (**I**) were tested. Bcl-2 expression (vs. Tubulin) and AKT phosphorylation (vs. AKT1) was quantified (D and G). “Non” stands for un-transfected parental cells (H and I). “vehicle” stands for 0.1% of DMSO (A-C). Error bars indicate SD. *p<0.05 vs. untreated control (“Ctrl”) group. ^#^*p*<0.05 vs. “c-siRNA” group (E and F). ^#^*p*<0.05 vs. “Vector” group (H and I).

In this report, the possible PKC412's resistance factors were also analyzed. We focused on Bcl-2, which is a well-known anti-apoptosis protein [14, 15]. ABT-737, a pharmacological inhibitor of Bcl-2 family proteins [16, 17], was applied. As demonstrated, co-treatment with ABT-737 in HT-29 cells dramatically potentiated PKC412's cytotoxicity, leading to profound cell death (Figure 5A) and apoptosis (Figure 5B). In primary cancer cells, co-administration of PKC412 and ABT-737 resulted in substantial cell death (Figure 5C), and the combined cytotoxicity was more potent than either single treatment (Figure 5C).

Since ABT-737 inhibits Bcl-2 as well as other Bcl-2 family member proteins [16, 17], we next utilized genetic strategies to alter Bcl-2 expression in CRC cells. First, siRNA was applied to selectively knockdown Bcl-2. Western blot assay results in Figure 5D confirmed that the two different Bcl-2 siRNAs (“-1 or -2”) both downregulated Bcl-2 in HT-29 cells. Intriguingly, PKC412's cytotoxicity was augmented in the Bcl-2-silenced cells (Figure 5E and 5F). Bcl-2 siRNA-2 [18] was more efficient in downregulating Bcl-2 than Bcl-2 siRNA-1 [19] (Figure 5D), and it was also more dramatic in sensitizing HT-29 cells to PKC412 (Figure 5E and 5F). Notably, ABT-737 (Figure 5A-5C) or Bcl-2 siRNA (Figure 5E and 5F) alone induced minor cytotoxicity and apoptosis activation in CRC cells. Based on the results above, we would speculate that Bcl-2 over-expression shall decrease PKC412's activity in CRC cells. Indeed, forced over-expression of Bcl-2 (Figure 5G), using a Bcl-2 expression vector ([20], see Methods), largely attenuated PKC412-induced viability reduction (Figure 5H) and apoptosis activation (Figure 5I) in HT-29 cells. These pharmacological and genetic evidences indicate that Bcl-2 could be a primary resistance factor of PKC412 in CRC cells.

### PKC412 inhibits HT-29 tumor growth *in vivo*

**Figure 6:**
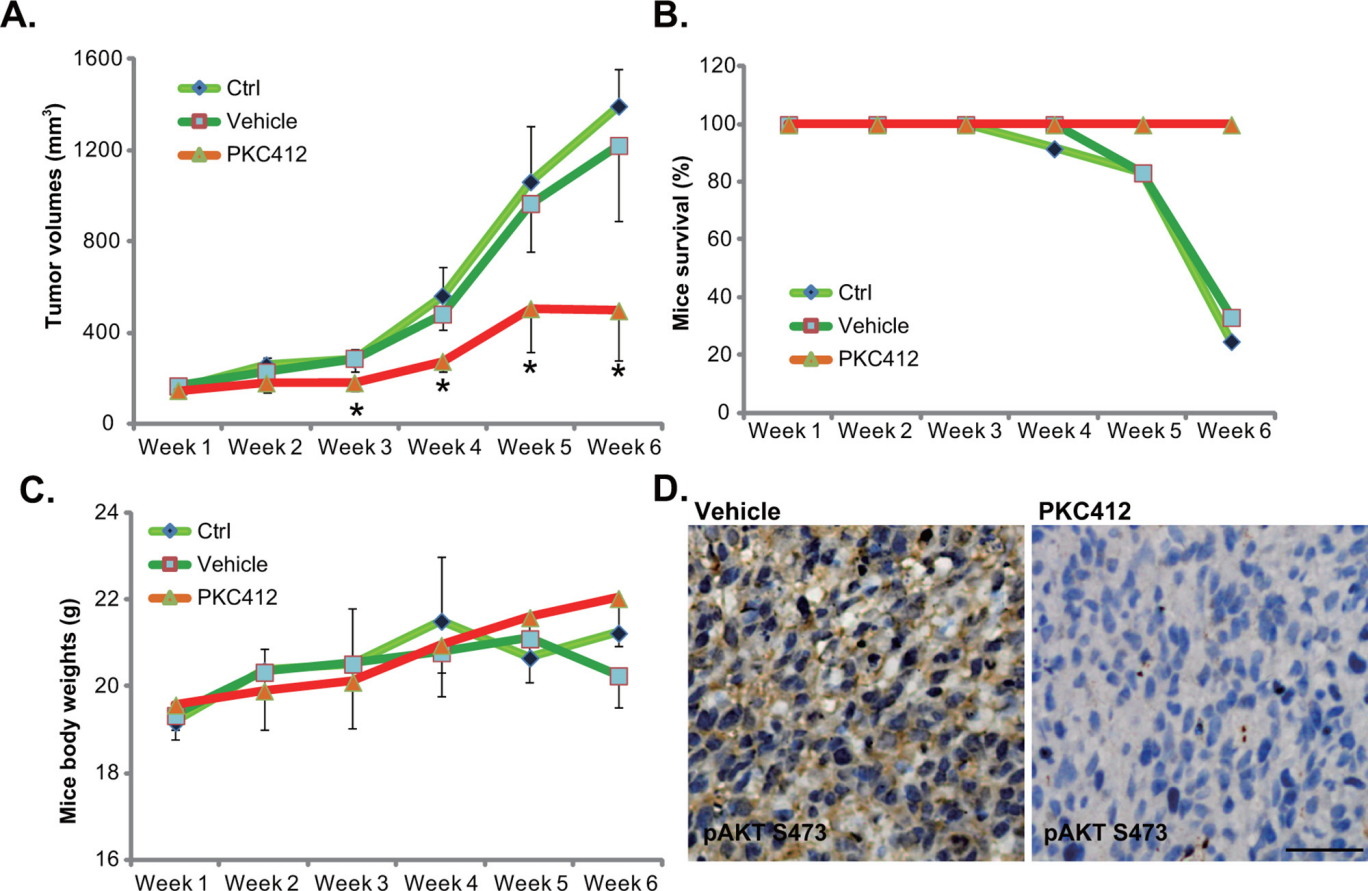
PKC412 inhibits HT-29 tumor growth *in vivo* HT-29 tumor-bearing nude mice were left untreated (“Ctrl”), or administrated with either PKC412 (100 mg/kg, oral gavage, daily) or the vehicle control (Gelucire 44/14, “Vehicle”), with 10 mice per group; Tumor volumes **A.**, mice survival **B.** and mice body weights **C.** were measured weekly for a total of 5 weeks (Week-1 to Week-6). Three days after initial PKC412 administration, HT-29 tumors were isolated, IHC was performed to test p-AKT (Ser-473), and representative images were presented **D.** *p<0.05 vs. “Ctrl” group (A). Bar=100 μm (D).

To study the anti-CRC activity by PKC412 *in vivo*, the nude mice HT-29 xenograft model was applied [6]. A sufficient number of HT-29 cells were inoculated into the nude mice. Weekly tumor growth curve results in Figure 6A demonstrated that PKC412 oral administration (100 mg/kg, daily) [10] remarkably inhibited HT-29 xenograft tumor growth in nude mice. HT-29 xenografts of PKC412-treated group were much smaller than that of the control group (Figure 6A). On the other hand, the vehicle control (Gelucire 44/14, “Vehicle”) [10] showed no significant effect on HT-29 xenograft growth (Figure 6A). Results in Figure 6B demonstrated that PKC412 oral administration dramatically improved the experimental mice survival. Notably, the mice body weight, which is the indicator of animals' general health, was not significantly changed between the groups (Figure 6C). Thus, mice were well-tolerated to the PKC412 administration. We also tested AKT activation in PKC412-treated tumor tissues. IHC staining assay results in Figure 6D confirmed that PKC412 administration significantly inhibited p-AKT in HT-29 tumors, which was consistent with the signaling results *in vitro* (Figure 4). Together, these results demonstrated that oral administration of PKC412 inhibits AKT and HT-29 tumor growth *in vivo*.

## DISCUSSION

The CRC's molecular heterogeneity hinders the uniform application of specific molecularly targeted-agent [2, 5]. Multiple kinase receptors, including PDGFR, VEGFR and Kit, were hyper-activated in CRC cells, which promote cancer cell survival, proliferation, migration, as well as apoptosis evasion and chemo-resistance [9]. Therefore, simultaneous blockage of multiple receptor kinases should achieve better anti-cancer efficiency than inhibition of each single kinase [2].

PKC412 is initially developed as a PKC inhibitor [7]. This compound could also block several pro-cancerous receptor kinases, including VEGFR-2, PDGFR-α, PDGFR-β, and the c-kit [8]. Here we showed that PKC412 inhibited survival and proliferation of both primary and established CRC cells. Meanwhile, PKC412 treatment led to apoptotic death and G2-M arrest in CRC cells. *In vivo*, oral administration of PKC412 inhibited HT-29 xenograft tumor growth in nude mice and improved mice survival. These results implied that PKC412 could be further tested as a promising anti-CRC agent.

AKT hyper-activity in CRC promotes cancer cell progression [12]. Thus, AKT is an known oncotarget for CRC [12]. Since PKC412 theoretically blocks multiple AKT upstream receptor kinases [8], it is not surprised to see that AKT activation was inhibited by PKC412 *in vitro* and *in vivo*. This could be the primary cause for subsequent cytotoxicity by this compound. Indeed, expression of CA-AKT1 largely attenuated PKC412's cytotoxicity. It should be noted that basal AKT activation is quite low in the non-cancerous epithelial cells, that might explain why these cells were not targeted by this multi-kinase inhibitor.

An important finding of the study is that Bcl-2, a known anti-apoptosis protein [15, 21], could be the primary resistance factor of PKC412 in CRC cells. We showed that Bcl-2 inhibition (by ABT-737 [17]) or siRNA knockdown dramatically augmented PKC412-induced CRC cell death and apoptosis. Reversely, PKC412's cytotoxicity was decreased in CRC cells with Bcl-2 over-expression. Further studies will be needed to explore the mechanistic insights of Bcl-2's inhibition on PKC412's activity, and if this could also be seen in other cancer cells.

## MATERIALS AND METHODS

### Chemicals and reagents

PKC412 was purchased from Shanghai Lan-jun Biotechnology (Shanghai, China). The Bcl-2 inhibitor ABT-737 was obtained from Selleck (Shanghai, China). Caspase inhibitors z-VAD-CHO and z-DEVD-CHO were obtained from Sigma Chemicals (Shanghai, China). All antibodies utilized were obtained from Cell Signaling Tech (Denver, MA).

### Culture of established cell lines

Human CRC cell lines, including HT-29, HCT-116, HT-15 and DLD-1, were described previously [6]. Cells were maintained in DMEM/RPMI medium, supplemented with 10% fetal bovine serum (FBS) and necessary antibiotics. All culture reagents were obtained from Sigma.

### Culture of primary cells

Isolation, preparation and culture of human colon cancer cells and colon epithelial cells were described previously [6, 22]. Briefly, cancer issues and surrounding normal epithelial tissues from informed-consent patients were minced, washed, and digested. Resolving single cell suspensions were pelleted, and resuspended in primary cell culture medium [6]. The protocol requiring human specimens was approved by the institutional review board and ethics board of all authors' institutions. All investigations were conducted according to the principles expressed in the Declaration of Helsinki.

### Cell viability MTT assay

As described previously [6], after treatment, the cell survival was measured through the routine 3-[4,5-dimethylthylthiazol-2-yl]-2,5 diphenyltetrazolium bromide (MTT) (Sigma) assay using the attached protocol [6].

### “Dead” cell detection by trypan blue staining

As described [6], after treatment, the number of dead CRC cells with positive trypan blue staining was counted automatically via a handheld cell counter (Merck Millipore, Shanghai, China).

### Clonogenicity assay

Following the designated PKC412 treatment,HT-29 cells (5 × 10^3^ per treatment) were suspended in 1 mL of DMEM containing 0.25% agar (Sigma), which was then added on top of a pre-solidified 100 mm culture dish. After 10 days of incubation, proliferative colonies were manually counted.

### Annexin V FACS analysis

Following the designated PKC412 treatment, cell apoptosis was tested by the Annexin V In Situ Cell Apoptosis Detection Kit (Roche, Indianapolis, IN) [6, 10]. Briefly, cells were washed and stained with Annexin V (Roche) and propidium iodide (PI) (Roche). The cell apoptosis ratio was reflected by Annexin V^+/+^/PI^−/−^ plus Annexin V^+/+^/PI^+/+^ percentage detected by fluorescence-activated cell sorting (FACS) (Beckman Coulter, Shanghai, China).

### Quantification of apoptosis by enzyme-linked immunosorbent assay (ELISA)

As described in our previous studies [6, 11], the Cell Apoptosis Histone-DNA ELISA Detection Kit (Roche, Palo Alto, CA) was applied to quantify cell apoptosis. ELISA OD was recorded as a quantitative indicator of cell apoptosis.

### Caspase activity assay

After applied PKC412 treatment in CRC cells, cytosolic extracts were added to caspase assay buffer containing caspase-3/-9 substrate [6, 10]. After 2 hours incubation, the release of AFC was quantified at an excitation value of 355 nm and emission value of 525 nm.

### Cell cycle analysis

After treatment with applied PKC412, CRC cells were trypsinized and fixed. Subsequently, cell suspension was incubated with 20 μL DNase-free RNase (10 mg/mL) and 1 mL of DNA intercalating dye PI (50 μg/mL, Triton-X 100 1.0%) at 4 °C for 30 min. Cell cycle phase analysis was performed by flow cytometry using the FACS machine [6].

### Western blot assay

Cells were washed with ice-cold PBS, and then lysed [10]. Protein lysates were separated on 10-12% SDS-PAGE gel, and were transferred to polyvinylidene fluoride (PVDF) membranes (Millipore, Shanghai, China), which were then blocked, incubated with primary and second antibodies. The detection of antigen-antibody binding was performed by Super-signal West Pico Enhanced Chemiluminescent (ECL) Substrates. The band intensity was quantified as described [6, 10].

### Constitutively active AKT1 (CA-AKT1) transfection

The plasmid encoding a constitutively active AKT1 (CA-AKT1) as well as the empty vector (“Ad-GFP”) were described in our previous study [6]. Cells were seeded onto 6-well plate with 60-70% confluence. Lipofectamine 2000 (Invitrogen, Shanghai, China) was applied to transfected the CA-AKT1 or the empty vector with recommended procedure [23]. Stable cells were selected by puromycin (2.5 μg/mL, Sigma).

### siRNA knockdown of Bcl-2

siRNA sequences for human Bcl-2 were designed as 5′-GCUGCACCUGACGCCCUUCtg-3′ (Bcl-2 siRNA-1) [19] and (5′-GCCCUGAUUGUGUAUAUUCA-3′ (Bcl-2 siRNA-2) [18], and were synthesized by Suzhou Genepharm (Suzhou, China). A negative control scramble siRNA was purchased Santa Cruz Biotech. siRNA (200 nM) transfection was performed through Lipofectamine 2000. The transfection took 36 hours. The efficiency of siRNA was verified via Western blot assay of Bcl-2.

### Forced Bcl-2 overexpression

The Bcl-2 expression construct in pSuper-puro plasmid was provided by Dr. Cui's group at Tianjin Medical University [20]. Lipofectamine 2000 was applied to transfect the Bcl-2 construct or the empty vector (pSuper-puro) to HT-29 cells. The stable cells were selected via puromycin (2.5 μg/mL). Bcl-2 expression in stable cells was always tested by Western blot assay.

### Xenograft assays

All animal procedures were performed according to the IACUC guidelines upon approval by all authors' institutional review boards. All investigations were conducted according to the NIH regulations. Athymic nude mice (C57BL/6 J background) were purchased from Suzhou University Institute of Biological Science. Mice were housed under standard conditions (12 hour-light/12 hour-dark at 21~23°C and 60~85% humidity) with ad libitum access to sterilized food and water. HT-29 cells (2×10^6^ cells in 100 μL of saline/Matrigel, 1:1 v/v) [10] were injected subcutaneously into the right flanks of 4-weeks-old female mice. PKC412 (6% w/w in Gelucire 44/14, Gattefosse, France) was stored at 4°C as a waxy solid formulation [10]. Treatments were started after tumor reached approximately 100 mm^3^ (around 2 weeks following inoculation). Prior to administration, the GC/PKC412 mixture was melted in a 45°C water bath and diluted with sterilized deionized water. The animals were weighed on a regular basis to ensure that a consistent dose (100 mg/kg/day [10]) of PKC412 was administered. Dosing was performed daily by oral gavage for a total of 21 days. Vehicle control animals received the same volume of GC solution. The size of the tumors was measured by caliper every week, and tumor volume was calculated using the following formula: π/6×width^2^× length [10]. Body weights and mice survival were also recorded every week [6].

### Immunohistochemistry (IHC) staining

The detailed protocol for IHC staining was described in other study [24]. Briefly, the staining was performed on cryostat sections (4 μm/section) of HT-29 xenograft tumors [25]. The slides were incubated with the primary antibody (anti-AKT Ser-473, 1: 100), and subsequently stained with horseradish peroxidase (HRP)-coupled secondary antibody (Santa Cruz). The slides were then visualized via peroxidase activity.

### Statistical analysis

The data presented in this study were means ± standard deviation (SD). Statistical differences were analyzed by one-way *ANOVA* followed by multiple comparisons performed with post hoc Bonferroni test (SPSS version 18). Values of *p* < 0.05 were considered statistically significant.
